# Unveiling the multitarget mechanism of Liuwei Dihuang decoction in autism spectrum disorder via network pharmacology and molecular docking

**DOI:** 10.1038/s41598-025-28204-1

**Published:** 2025-11-25

**Authors:** Zilin Chen, Xu Wang, Fei Han

**Affiliations:** 1 Department of Pediatrics, Guang’anmen Hospital, China Academy of Chinese Medical Sciences, Beijing, China; 2 State Key Laboratory of Traditional Chinese Medicine Syndrome, The Second Affiliated Hospital of Guangzhou University of Chinese Medicine, Guangzhou, Guangdong China

**Keywords:** Autism spectrum disorders, Liuwei Dihuang decoction, Network pharmacology, Microarray data, Molecular docking, Molecular dynamics simulations, Pharmacology, Computational biology and bioinformatics, Neuroscience

## Abstract

Liuwei Dihuang decoction (LW) is used for paediatric autism spectrum disorders (ASD) treatment, but its mechanism of action is unclear. This study aims to provide clinical evidence for LW in treating ASD in children and investigate its mechanism through network pharmacology, microarray analysis, and molecular docking. A retrospective review of 80 clinical cases of children with ASD and the therapeutic effect of LW was conducted. Drug-disease co-expressed genes were used to construct PPI network maps. The active ingredients of LW were obtained from the TCMSP, CNKI, and PubMed, with screening criteria of OB ≥ 30% and DL ≥ 0.18. Disease targets were sourced from the GeneCards, OMIM, and DisGeNET databases. Core targets were further analyzed using GO and KEGG. Microarray data were employed to analyze the expression levels of the core targets. Molecular docking and dynamics simulations were performed on protein–ligand complexes. MD simulations were performed using GROMACS 2022 for 100 ns, and the stability of the complex was evaluated by analyzing key parameters including RMSD, RMSF, and hydrogen bond occupancy. LW showed promising therapeutic effects on ASD, with efficacy decreasing with age. Quercetin, Tetrahydroalstonine, Diosgenin and Kaempferol were identified as active compounds. PTGS2 and MMP9 were upregulated and identified as key genes in ASD treatment. The binding energies of the key complexes PTGS2-Quercetin, PTGS2-Tetrahydroalstonine, MMP9-Diosgenin, and MMP9-Kaempferol were determined to be − 9.5 kJ/mol, − 9.4 kJ/mol, − 8.1 kJ/mol, and − 7.2 kJ/mol, respectively. Molecular simulations showed favorable binding between key genes and active compounds. This study provides clinical evidence for the treatment of ASD with LW, and predicts its main active ingredients, potential pathways, and core targets, providing a reliable basis for the clinical treatment and drug screening of ASD.

**Supplementary Information:**

The online version contains supplementary material available at 10.1038/s41598-025-28204-1.

## Introduction

Autism Spectrum Disorder (ASD), also known as autism, is a generalized neurodevelopmental disorder that typically emerges in infancy and early childhood, and is more prevalent in males, with a male-to-female ratio of approximately 4:1^1^. Clinically, the main manifestations include varying degrees of language developmental disorders, interpersonal difficulties, narrow interests, and repetitive stereotyped behaviors^2^. With the incidence of ASD increasing annually, it has become a significant challenge in global public health^3^. In China, there are over 10 million individuals with ASD, including more than 2 million children, resulting in an ASD incidence rate of over 1%^4^. However, the full pathogenesis of ASD has not been fully elucidated, although some studies have indicated that ASD is closely linked to genetic and environmental factors, including mutations in specific genes and genetic susceptibility. It is estimated that more than 800 genes and numerous genetic syndromes are associated with ASD^5^, while environmental factors such as exposure to certain drugs, infections, and nutritional deficiencies during pregnancy also contribute to the development of ASD^6^. Currently, in the absence of a clear etiology and pathogenesis of ASD, medication management remains challenging, and behavioral rehabilitation training is often the primary treatment^7^. Several psychotropic medications with distinct mechanisms of action have been approved for alleviating ASD-related symptoms and comorbidities. For instance, aripiprazole, risperidone, and haloperidol are indicated for irritability and aggressive behaviors^8^, while methylphenidate, atomoxetine, clonidine, and guanfacine are used to manage hyperactivity^9^. Additionally, melatonin has been employed to address sleep disturbances^10^. However, they have relatively high side effects and are prone to relapse after discontinuation^11^. Therefore, the limited therapeutic options for ASD have garnered significant attention from researchers, emphasizing the importance of finding drugs with mild pharmacological properties and targeted effects.

Chinese herbal medicine is recognized for its minimal adverse effects in the treatment of diseases. Liuwei Dihuang decoction (LW) is a traditional Chinese herbal formula derived from the ancient Chinese medical text “Key to Diagnosis and Treatment of Children’s Diseases”. It has been widely utilized to address a range of disorders stemming from renal yin insufficiency. Modern pharmacological studies have revealed that LW possesses multiple neuropharmacological activities, including anti-neuroinflammation^12^, anti-neurooxidation^13^, and neuroprotection^14^, potential therapeutic efficacy in the treatment of neurological disorders such as cognitive impairment^15^. However, studies on LW for ASD are limited, and its therapeutic mechanism requires further exploration.

Network pharmacology is an emerging research methodology that integrates bioinformatics, systems biology, and pharmacology to elucidate drug action mechanisms and identify potential therapeutic targets by constructing drug-target-disease networks. In this study, we will review 80 children with ASD and evaluate the therapeutic effects of LW on them. Then, we will employ network pharmacology and microarray data analysis to develop multi-component and multi-target models aimed at screening and predicting the primary active compounds, potential pathways, and targets of LW for ASD. Subsequently, we will simulate and analyze the conformational changes, stability, and interaction mechanisms of target proteins and small molecule complexes through 100 ns molecular dynamics (MD) simulations and MMPBSA binding free energy analysis. This comprehensive approach will provide insights into the mechanism of action of LW in the treatment of ASD.

## Materials and methods

### The clinical efficacy evaluation of LW

#### Source of ASD cases

This study collected 80 cases of ASD children who were treated at the Guang’anmen Hospital of the China Academy of Chinese Medical Sciences from June 2022 to June 2024.

#### Ethical statement

This retrospective study was conducted in accordance with the Declaration of Helsinki and was granted an exemption from ethical review by the Ethics Committee of Guang’anmen Hospital, China Academy of Chinese Medical Sciences. The requirement for informed consent from parents was waived by the same committee because this retrospective study analyzed anonymized, routinely collected clinical data with no personally identifiable information.

#### ASD diagnostic criteria

Diagnosis tools: ABC, Autism Behavior Checklist; CARS, Childhood Autism Rating Scale; DSM-V, Diagnostic and Statistical Manual of Mental Disorders, 5th edition.

#### Inclusion criteria

① Meet the diagnostic criteria for ASD; ② Age ≥ 2 years; ③ No psychotropic drugs targeting the core symptoms of ASD (such as risperidone, aripiprazole) have been taken since the onset of the disease, but those taking basic nutritional supplements or having received behavioral rehabilitation training are not excluded; ④ Good compliance, willing to accept 6 months of treatment.

#### Exclusion criteria

① Children with comorbid schizophrenia or other related mental disorders; ② Those who have already received treatment related to autism; ③ Those with severe cardiovascular or physical illnesses; ④ Parents of the child who refuse to acknowledge the child’s condition or cooperate in filling out scales.

#### Exit criteria

① Patients who are uncooperative and fail to complete the scales as required; ② Those who do not take medication as required or have discontinued medication for more than 1 week; ③ Patients’ parents who request to withdraw from the study.

#### Clinical efficacy evaluation criteria

Therapeutic efficacy index (%) = (pre-treatment score − post-treatment score)/pre-treatment score × 100%. Treatment effectiveness is considered when the efficacy index is > 5%.

### Data collection

#### Virtual screening of active compounds in LW

Data on compounds rehmannia rhizome (SDH), cornus (SZY), common yam rhizome (SY), poria (FL), tree peony bark (DP), and alisma rhizome (ZX) in LW were collected from the Traditional Chinese Medicine Systematic Pharmacology Database and Analysis Platform (TCMSP, https://tcmsp-e.com/). Additional information on other compounds was also obtained from CNKI (https://www.cnki.net/) and PubMed (https://pubmed.ncbi.nlm.nih.gov/). The screening criteria for bioactive compounds included an oral bioavailability (OB) of ≥ 30% and a drug likeness (DL) value of ≥ 0.18.

#### Finding potential therapeutic targets for LW

The target protein information of the active compounds in LW was searched using the TCMSP-related target module, and the target protein was standardized in the Uniport database (https://www.uniprot.org), with the species restricted to “Human”, the search results restricted to verified, and finally obtain the corresponding gene name of the target protein.

#### Retrieving and integrating ASD-related genes from existing databases

The GeneCards database (http://www.genecards.org/), the OMIM database (http://www.omim.org/), and the DisGeNET database (https://www.disgenet.org/) were searched for disease genes associated with ASD using “autism spectrum disorder” as the keyword. The search results from these three databases were finally combined to obtain ASD-related disease targets after removing duplicate values.

#### Identification of drug-disease co-expression targets

The collected therapeutic targets of LW and ASD targets were analyzed using the Venny 2.1.0 online platform (http://www.liuxiaoyuyuan.cn/) to identify common targets between the drug and the disease. These shared genes were considered as potential effective targets for LW in the treatment of autism.

### Construction of PPI network diagram

The drug-disease common targets that were inputted into the STRING database (https://string-db.org) with the species restricted to “Homo sapiens” and a confidence score threshold set at ≥ 0.4. This allowed us to obtain protein–protein interaction data. The obtained data was then imported into Cytoscape 3.10.1 software for visualization and analysis, resulting in the construction of a Protein–Protein Interaction (PPI) network diagram.

### Topology analysis and cluster analysis of PPI networks

Topology analysis and clustering analysis of the PPI networks were carried out using the CytoNCA plug-in. Degree, Between Centrality (BC), and Closeness Centrality (CC) were employed as the common evaluation methods for the core nodes of the network. Core target points were determined using a screening criteria where Degree, BC, and CC values were all greater than the median. This method was applied twice to finalize the core target points.

### Network construction

Cytoscape 3.10.1 software was utilized to construct the network diagram of active ingredient-target interactions. In this diagram, “nodes” represent the active ingredients and their targets, while “edges” depict the interactions between the active ingredients and the targets. The final visualized network diagram comprises both “nodes” and “edges”.

### Functional annotation and pathway analysis

To explore the core mechanisms and pathways associated with all potential targets, Gene Ontology (GO) functional enrichment analysis and Kyoto Encyclopedia of Genes and Genomes (KEGG) pathway enrichment analysis were performed. The Metascape database (http://metascape.org/) was employed to conduct these analyses for the key targets identified earlier. The GO functional enrichment analysis encompassed three main aspects: Biological Process (BP), Cellular Component (CC), and Pathway Enrichment. On the other hand, KEGG pathway analysis provided insights into the higher functions and roles of biological systems. For further analysis, the top 20 GO-enriched pathways with high counts and the top 10 KEGG pathways with high counts were selected. Finally, the important GO terms and KEGG pathways can be visualized using the ggplot2 software package in R.

### Microarray data analysis

To validate the findings of this study, we obtained four microarray datasets (GSE28475, GSE28521, GSE38322, and GSE236761) from the Gene Expression Omnibus (GEO) database (https://www.ncbi.nlm.nih.gov/geo/) (Table 1). These datasets were analyzed separately to avoid confounding effects arising from different experimental platforms and batches. The “Limma” package in R was used to perform background correction and normalization for each dataset independently. Subsequently, differentially expressed genes (DEGs) were identified using the thresholds |logFC|≥ 1 and an adjusted *P* value (FDR) < 0.05. Volcano plots were then generated with the ggplot2 package in R to visually represent the significant and non-significant genes. Following this, the DEGs from all datasets were pooled and compared with the hub genes, and any commonalities between the DEGs and hub genes were selected for further analysis.

**Table 1:** GEO database data source.

Dataset	Platform	Control	Affected	Total	Source
GSE28475	GPL6883	61	52	113	Postmortem brain
GSE28521	GPL6883	40	39	79	postmortem brain
GSE38322	GPL10558	18	18	36	postmortem brain
GSE236761	GPL16791	8	11	19	postmortem brain

### Molecular docking analysis

Molecular docking was employed to identify the interactions between the target genes and the active compounds. The active compound components were obtained from the PubChem database (pubchem.ncbi.nlm.nih.gov/). Protein structures of the target genes were acquired from the RCSB Protein Data Bank (PDB) (https://www.rcsb.org/), which serves as a repository of biomolecule atomic coordinates and other relevant information. The protein structures obtained from the PDB were refined using PyMol software. Subsequently, the online tool Proteins Plus (https://proteins.plus/) was utilized to predict protein binding sites and identify the binding pockets of the target proteins. Molecular docking and virtual screening of the core targets and active compounds were performed using AutoDock software. Docking complexes with low root mean square deviation (RMSD) and low binding energies were selected for further analysis. The docking fraction represents the energy required for a chemical reaction to occur between reactants, and a lower docking fraction indicates a more stable and effective binding between molecules, resulting in a better binding affinity. Finally, the interaction of docking complexes was visualized in 3D and 2D using PyMol and Discovery Studio software.

### Molecular dynamics (MD) simulations and binding free energy analysis

The two ligand molecules with the smallest docking fractions in the core target proteins were selected for MD simulations and binding free energy analyses. The MD simulations were conducted using the Gromacs2022 program with the GAFF force field for small molecules and the AMBER14SB force field and TIP3P water model for proteins. The protein and small-molecule ligand files were merged to construct a simulation system for the complexes. The system was first energy-minimized using the steepest descent algorithm to remove any steric clashes. Then, the system was neutralized by adding an appropriate number of counterions (Na+/Cl−). The simulation was carried out at constant temperature and pressure under periodic boundary conditions. During MD simulations, all involved hydrogen bonds were constrained using the LINCS algorithm with an integration step of 2 fs. Electrostatic interactions were calculated using the Particle-mesh Ewald (PME) method with the cutoff value set to 1.2 nm. The non-bonded interactions cutoff value was set to 10 Å and updated every 10 steps. The V-rescale temperature coupling method was used to control the simulation temperature at 298 K, and the Berendsen method was used to control the pressure at 1 bar. Equilibrium simulations of NVT and NPT were conducted for 100 ps at 298 K. MD simulations were performed for 100 ns for the complex system, with conformations saved every 10 ps. After the simulations, the trajectory data was analyzed using VMD and PyMOL. The stability of the protein–ligand complex was assessed by conducting MMPBSA binding free energy analysis between the protein and the small molecule ligand using the g_mmpbsa program.

#### Statistical methods

Data analysis was performed using GraphPad Prism 9 statistical software. All statistical analyses were considered not statistically significant when *P* > 0.05, and statistically significant when *P* < 0.05. The data were first subjected to a normality test. For data that followed a normal distribution and had equal variances, a paired sample t-test was used. For data that did not follow a normal distribution, non-parametric tests were used.

## Results

### Clinical efficacy evaluation of LW

**Fig. 1:**
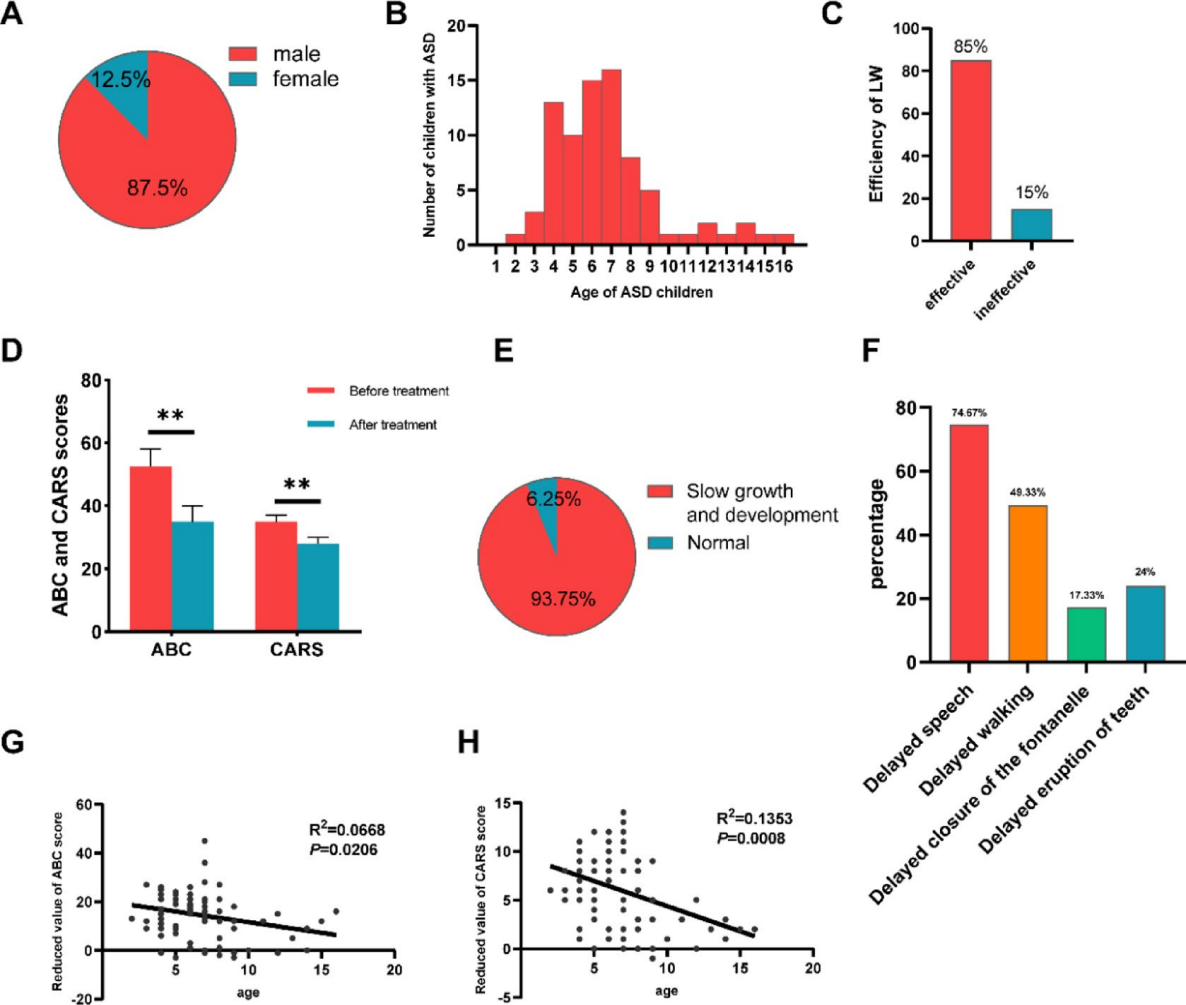
Clinical efficacy evaluation of LW. (**A**) Ratio of male to female ASD children. (**B**) Age distribution of children with ASD. (**C**) Proportion of children with ASD effectively treated with LW. (**D**) Changes in ABC and CARS scores after LW treatment. (**E**–**F**) Proportion of children with ASD who have developmental delays. (**G**-**H**) The correlation between age and changes in ABC and CARS scores.

We conducted a study on the efficacy of LW in 80 children with ASD, with 87.5% being male and 12.5% being female (Fig. 1A). The age distribution of the patients was mainly between 4 and 9 years old (Fig. 1B). After 6 months of LW treatment, 85% of the patients showed symptom improvement (Fig. 1C). We evaluated the severity of ASD using the ABC and CARS scales, and the results showed a significant decrease in scores in children after LW treatment, indicating the effectiveness of LW in treating ASD children (Fig. 1D). No significant adverse effects were reported or observed during the 6-month treatment period. LW was initially created to treat developmental delays in children, so we also assessed the developmental status of these children. We found that 93.75% of the children had developmental delays (Fig. 1E), with 74.67% experiencing delayed speech, 49.33% delayed walking, 17.33% delayed fontanel closure, and 24% delayed tooth eruption (Fig. 1F). Furthermore, we conducted a correlation analysis between age and changes in ABC and CARS scores before and after treatment. The results showed that the older the child, the less the decrease in scores, indicating slower symptom relief and reduced sensitivity to LW, leading to poor treatment outcomes (Fig. 1G, H). Therefore, early intervention and treatment are crucial for the development of children with ASD, helping them better adapt to social and learning environments and improve their quality of life.

### Active compounds and potential targets of LW

A total of 47 compounds were sourced from the TCMID database and literature for the six herbs of LW, with varying numbers of compounds in SDH, SZY, SY, FL, DP, and ZX, totaling 2, 14, 12, 6, 6, and 7 compounds, respectively. Notably, MOL000449 (stigmasterol) and MOL000359 (sitosterol) were identified as components present in SDH, SZY, and SY, with MOL000359 also being a common component of SDH, SZY, and DP. After removing duplicate components, a total of 42 unique compounds were identified and tabulated in Supplementary Table. The active compounds and their corresponding target information were then sequentially collected through the TCMSP target module, resulting in a total of 204 relevant targets after de-duplication.

### Component-target network diagram for LW

**Fig. 2:**
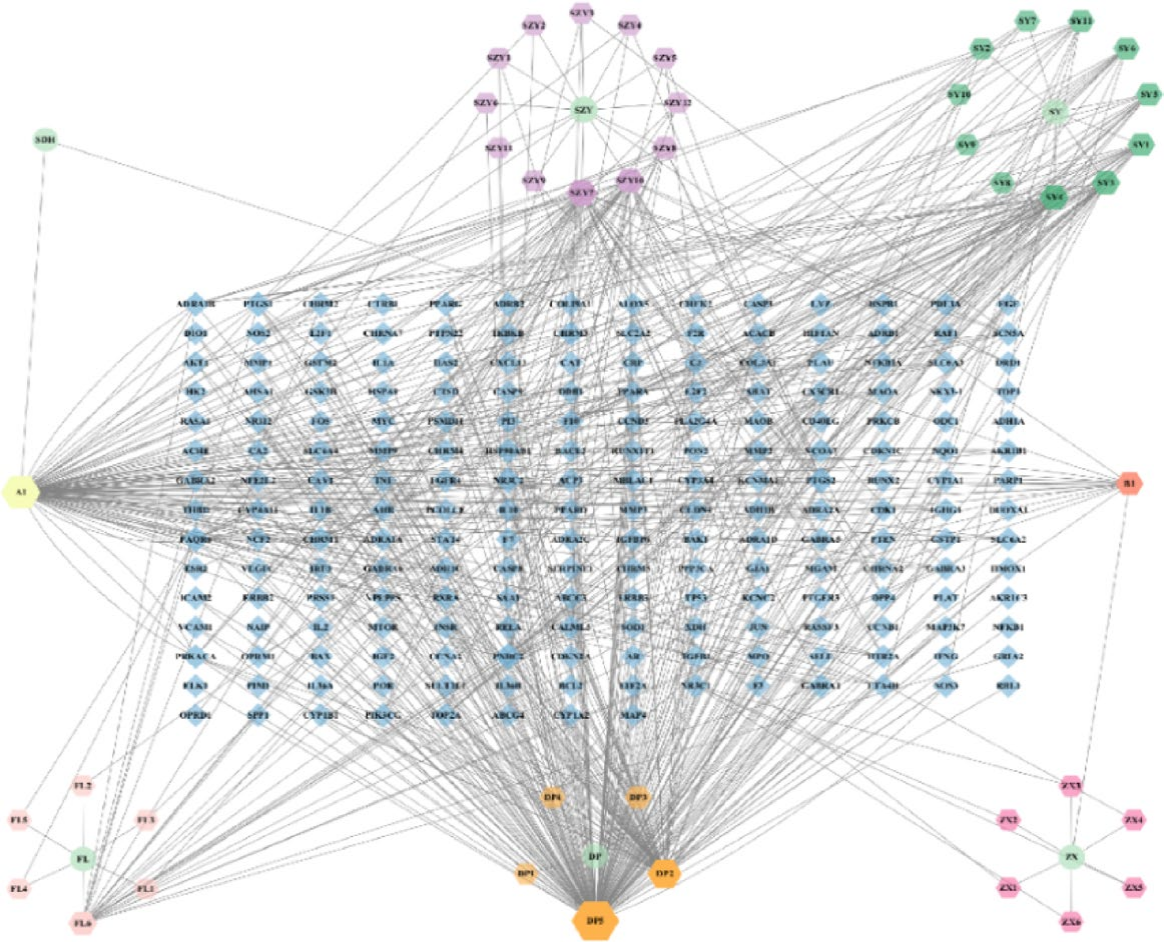
Component-target network diagram for LW.

The 42 active compounds and 204 targets were imported into Cytoscape 3.10.1 to generate the “active ingredient-target” network graph for LW in treating ASD. The network graph consisted of 252 nodes and 622 edges (Fig. 2). To identify the core chemical components of LW for ASD treatment, the network was analyzed using the Analyze Network function. The results revealed the top ten active chemical components, ranked by their degree centrality, as follows: 5280343-quercetin (degree: 152), 5280794-stigmasterol (degree: 96), 5280863-kaempferol (degree: 63), 222284-beta-sitosterol (degree: 39), 72340-tetrahydroalstonine (degree: 29), 122159-kadsurenone (degree: 28), 73299-hederagenin (degree: 24), 6443896-hancinone C (degree: 23), 99474-diosgenin (degree: 17), and 222284-sitosterol (degree: 16) (Table 2). Notably, Quercetin and Kaempferol are both flavonoids, Stigmasterol, Beta-sitosterol, Diosgenin and Sitosterol can be categorized as steroids, and Kadsurenone and Hancinone C are lignans. Each active ingredient has multiple targets, suggesting that the active targets of LW may exert synergistic effects in the treatment of ASD.

**Table 2:** The degree and class of 10 bioactive constituents examined through Cytoscape.

Compound	Class	Degree	Betweenness	Closeness
Quercetin	Flavonoids	152	0.640825435	0.560538117
Stigmasterol	Steroids	96	0.101540393	0.384615385
Kaempferol	Flavonoids	63	0.132911689	0.411184211
Beta-sitosterol	Steroids	39	0.088209023	0.386996904
Tetrahydroalstonine	Indole alkaloid	29	0.046520423	0.364431487
Kadsurenone	Lignanoids	28	0.044363646	0.369822485
Hederagenin	Triterpenoids	24	0.054937235	0.367647059
Hancinone C	Lignanoids	23	0.025205544	0.362318841
Diosgenin	Steroid Saponins and its Sapogenins	17	0.04981404	0.356125356
Sitosterol	Other Steroids	16	0.0250361	0.356125356

### Potential targets for LW in the treatment of ASD

**Fig. 3:**
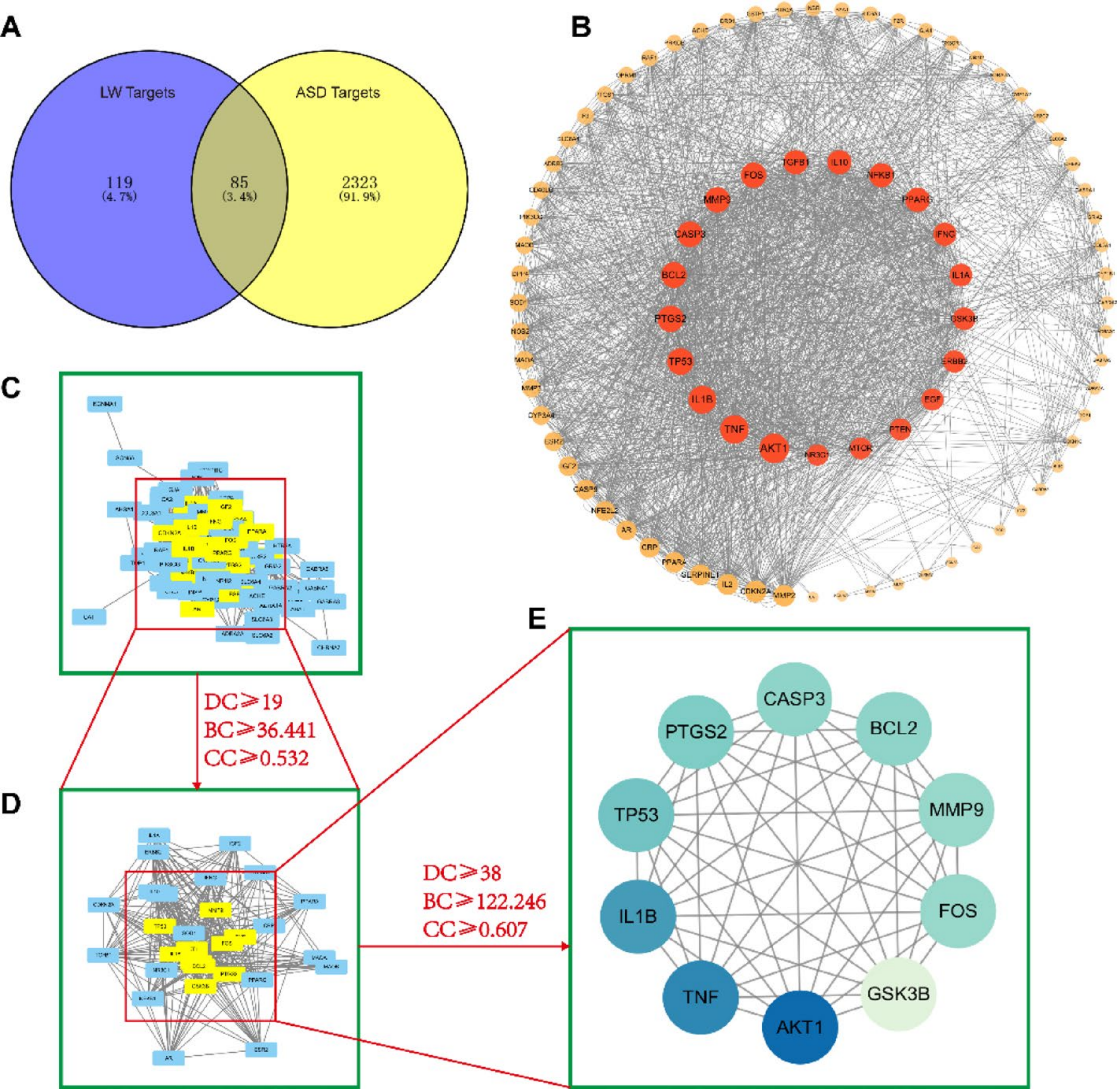
The Potential Target of LW in Treating ASD. (**A**) Wayne diagram of common target of LW active components and ASD. (**B**) Protein interaction network diagram. (**C**–**E**) Topology screening process for PPI networks.

The GeneCards, DisGeNET, and OMIM databases were queried using the keyword “autism spectrum disorder”, resulting in the identification of 2,323 ASD therapeutic targets after duplicates removal. Subsequently, the intersection of LW drug targets and ASD disease targets was conducted using Venny2.1.0 software, leading to the identification of 85 potential therapeutic targets (Fig. 3A).

### Construction of PPI protein interaction network

The 85 overlapping genes were utilized to generate a PPI protein interactions network map using the STRING database, which was then imported into Cytoscape 3.10.1. Subsequently, network analysis was conducted by CytoNCA, resulting in median values of 19, 36.441303, and 0.5316456 for DC, CC, and BC, respectively. The screening process was repeated with the criterion of greater-than-median, leading to the identification of 10 genes with high degree values, which may serve as key targets for disease treatment (Fig. 3B–E, Table 3).

**Table 3:** Information of 10 core targets.

NO	UniProt ID	Gene symbol	Protein name	degree
1	P31749	AKT1	RAC-alpha serine/threonine-protein kinase1	59
2	P01375	TNF	Tumor necrosis factor	56
3	P01584	IL1B	Interleukin-1 beta	54
4	P04637	TP53	Cellular tumor antigen p53	50
5	P35354	PTGS2	Prostaglandin G/H synthase 2	49
6	P42574	CASP3	Caspase-3	47
7	P10415	BCL2	Apoptosis regulator Bcl-2	47
8	P14780	MMP9	Matrix metalloproteinase-9	46
9	P01100	FOS	Proto-oncogene c-Fos	46
10	P49841	GSK3B	Glycogen synthase kinase-3 beta	38

### GO enrichment and KEGG pathway analysis

**Fig. 4:**
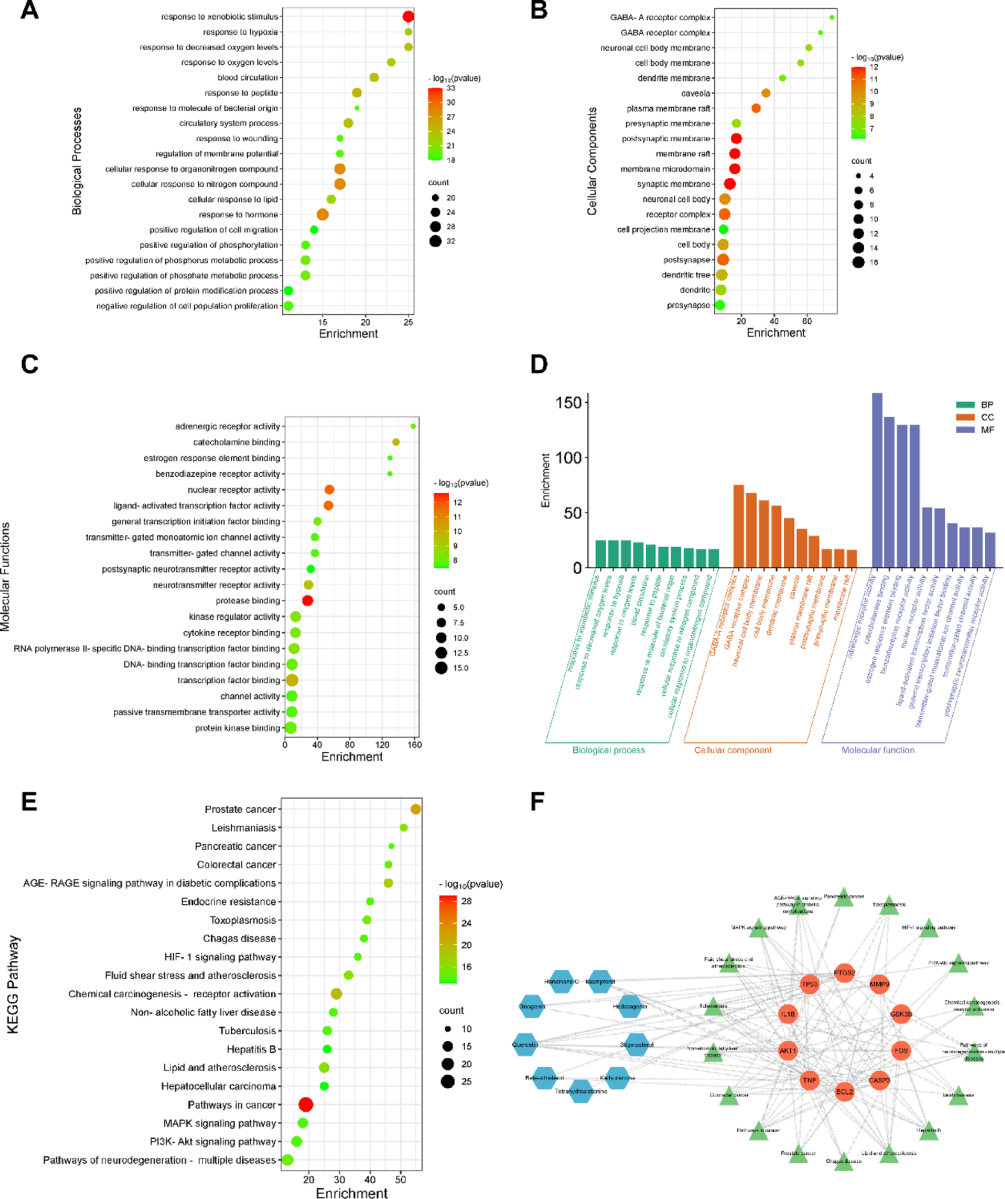
GO enrichment and KEGG pathway analysis. (**A**) Biological Process. (**B**) Cellular Component. (**C**) Molecular Function. (**D**) Top 10 for number of genes in each category. (**E**) KEGG pathway analysis. (**F**) Compound-target pathway network influenced by LW. The red nodes indicate the hub genes, the blue nodes indicate active constituents, and the green nodes are the pathways related to targets.

The 85 overlapping genes underwent GO functional annotation and enrichment analysis to investigate the biological functions of potential therapeutic targets of LW. A total of 1299 BP, 61 CC, 150 MF, and 180 KEGG pathways were enriched by the Metascape database. The significance threshold was set at a *P* value < 0.05, and the background gene list was the entire human genome provided by the Metascape platform. Based on the p-value and count value, the top 20 BP terms, CC terms, and MF terms of GO functional enrichment analysis were selected for visualization and represented as bubble plots (Fig. 4). The results indicated that the BP of LW potential targets might include xenobiotic stimulus, hormone, and nitrogen compound (Fig. 4A), etc. CC might include synaptic membrane, postsynaptic membrane, and membrane raft (Fig. 4B), etc. MF may include proteinase binding, nuclear receptor activity, and ligand-activated transcription factor activity, etc. (Fig. 4C). The top 10 items of the number of genes in each category were depicted as bar graphs (Fig. 4D). Furthermore, important signaling pathways associated with ASD were identified through KEGG pathway analysis. For instance, Pathways in cancer, Prostate cancer, Chemical carcinogenesis-receptor activation, AGE-RAGE signaling pathway in diabetic complications, and Leishmaniasis (Fig. 4E)^16–18^ were among the pathways identified. Additionally, KEGG pathway analysis revealed that AKT1, BCL2, CASP3, and TNF were significantly enriched genes involved in the function of several pathways, including pathways in cancer, Prostate cancer, Chemical carcinogenesis-receptor activation, and others (Fig. 4F).

### Microarray data analysis

**Fig. 5:**
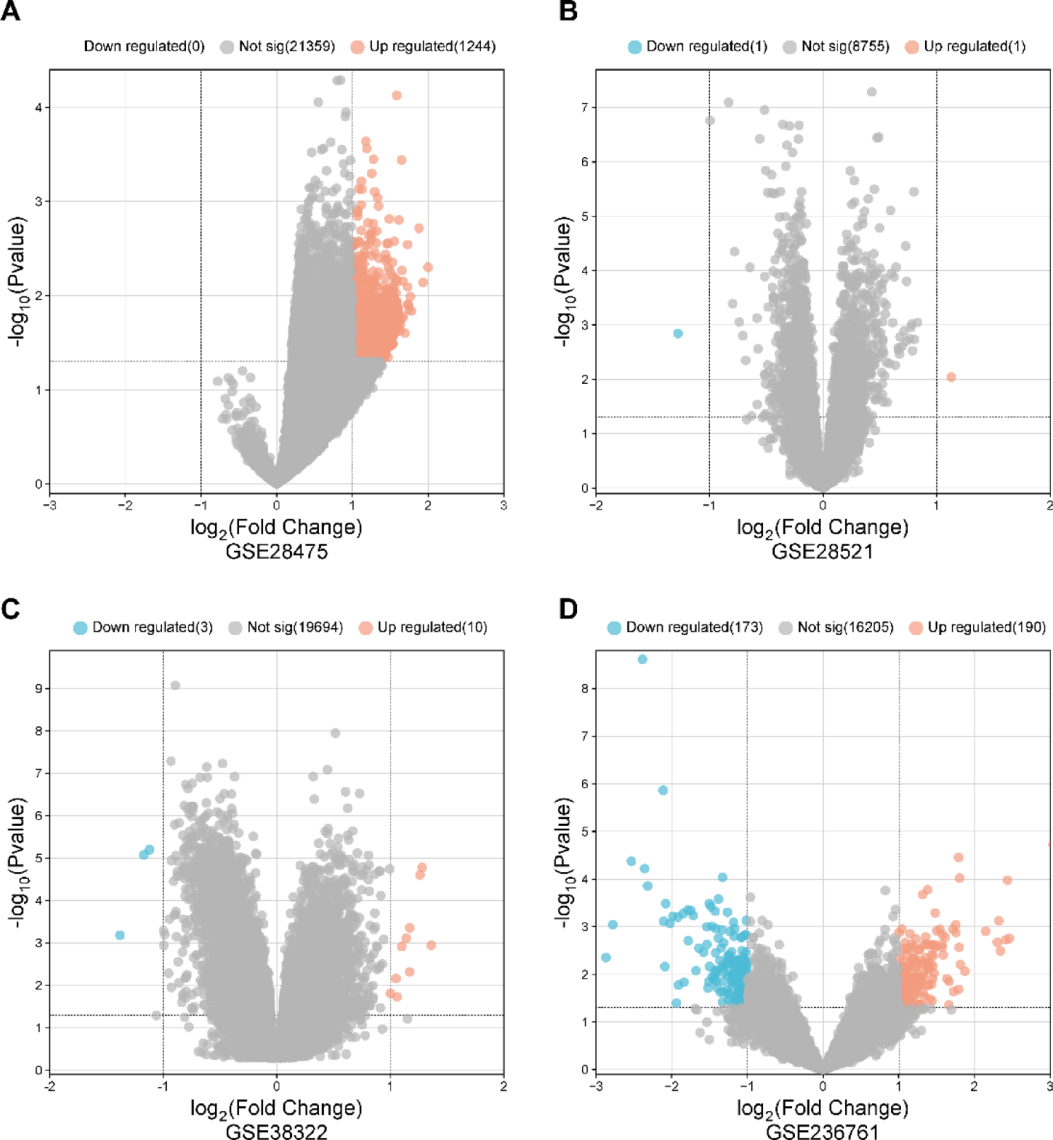
Volcano plot of (**A**) GSE28475, (**B**) GSE2852, (**C**) GSE38322, and (**D**) GSE236761 datasets. The red color represents genes that are upregulated, the blue color represents genes that are downregulated.

To further validate the identification of ASD-related targets, we conducted microarray data analysis. In GSE28475, 1244 genes were up-regulated (Fig. 5A), while in GSE28521, only 1 gene was up-regulated and 1 gene was down-regulated, which is likely due to the inherent homogeneity of the samples or the specific profiling platform used in this particular dataset (Fig. 5B). In GSE38322, 10 genes were up-regulated and 3 genes were down-regulated (Fig. 5C). In GSE236761, 190 genes were up-regulated and 173 genes were down-regulated (Fig. 5D). Across the four GEO datasets, a total of six differentially expressed genes with drug-disease targets were identified, namely F3, IL10, MAOA, MMP9, PPARG, and PTGS2. Among them, PTGS2 and MMP9 genes were found in the same 10 hub genes mentioned above, and both were identified in GSE28475. Consequently, PTGS2 and MMP9 were selected as target genes for molecular docking analysis.

### Molecular docking analysis

We conducted molecular docking of the PTGS2 (UniProt ID: P35354) and MMP9 (UniProt ID: P14780) genes with 10 major active compounds, including Quercetin, Stigmasterol, Kaempferol, Beta-sitosterol, Tetrahydroalstonine, Kadsurenone, Hederagenin, Hancinone C, Diosgenin, and Sitosterol, individually. The calculated values for PTGS2 were an Area (SA) of 3333.16 Å2 and a Volume (SA) of 3107.12 Å3, while for MMP9, an Area (SA) of 688.05 Å2 and a Volume (SA) of 506.18 Å3 were obtained. The predicted center coordinates of the docking boxes for PTGS2 and MMP9 in AutoDock were X: 23.92, Y: 45.863, Z: 61.059 and X: 22.618, Y: 11.159, Z: 43.723, respectively. The results revealed that Quercetin exhibited a strong affinity for PTGS2 with a binding energy of − 9.4 kJ/mol. Tetrahydroalstonine showed a binding energy of − 9.4 kJ/mol with a RMSD of 1.043 Å, and Kaempferol exhibited a binding energy of − 9.3 kJ/mol with a RMSD of 0.240 Å. These three compounds demonstrated a strong affinity and stability for PTGS2. Diosgenin and Hederagenin also displayed strong binding energies, ranking in the top five, with binding scores of − 8.9 kJ/mol and − 8.6 kJ/mol, respectively. For MMP9, Diosgenin exhibited a binding energy of − 8.1 kJ/mol with an RMSD of 0.265 Å, indicating a strong affinity and stability. Kaempferol displayed a binding energy of − 7.2 kJ/mol with an RMSD of 0.112 Å, demonstrating strong affinity and stability as well. Stigmasterol, Sitosterol, and Beta-sitosterol were among the top five compounds with binding energies of − 7.1 kJ/mol, − 7.0 kJ/mol, and − 7.0 kJ/mol, respectively (Table 4).

**Table 4:** Interaction and binding energy between proteins of target genes and active compounds.

Protein	Ligand	Binding affinity(kJ/mol)	RMSD
PTGS2	Quercetin	-9.5	0.256
	Tetrahydroalstonine	-9.4	1.043
	Kaempferol	-9.3	0.240
	Diosgenin	-8.9	0.382
	Hederagenin	-8.6	0.061
	Hancinone C	-8.5	0.241
	Kadsurenone	-8.4	0.951
	Stigmasterol	-8.2	0.517
	Sitosterol	-8.2	1.949
	Beta-sitosterol	-7.7	1.317
MMP9	Diosgenin	-8.1	0.265
	Kaempferol	-7.2	0.112
	Stigmasterol	-7.1	0.172
	Sitosterol	-7.0	0.504
	Beta-sitosterol	-7.0	0.959
	Kadsurenone	-6.9	1.116
	Tetrahydroalstonine	-6.9	1.154
	Hederagenin	-6.7	0.419
	Quercetin	-6.4	0.083
	Hancinone C	-5.9	0.464

### MD simulations and binding free energy analysis

The two ligand molecules with the lowest docking scores in PTGS2 and MMP9, namely PTGS2- Quercetin, PTGS2-Tetrahydroalstonine, MMP9- Diosgenin, and MMP9-Kaempferol, were chosen for MD simulations and binding free energy analysis.

#### Stabilization analysis

**Fig. 6:**
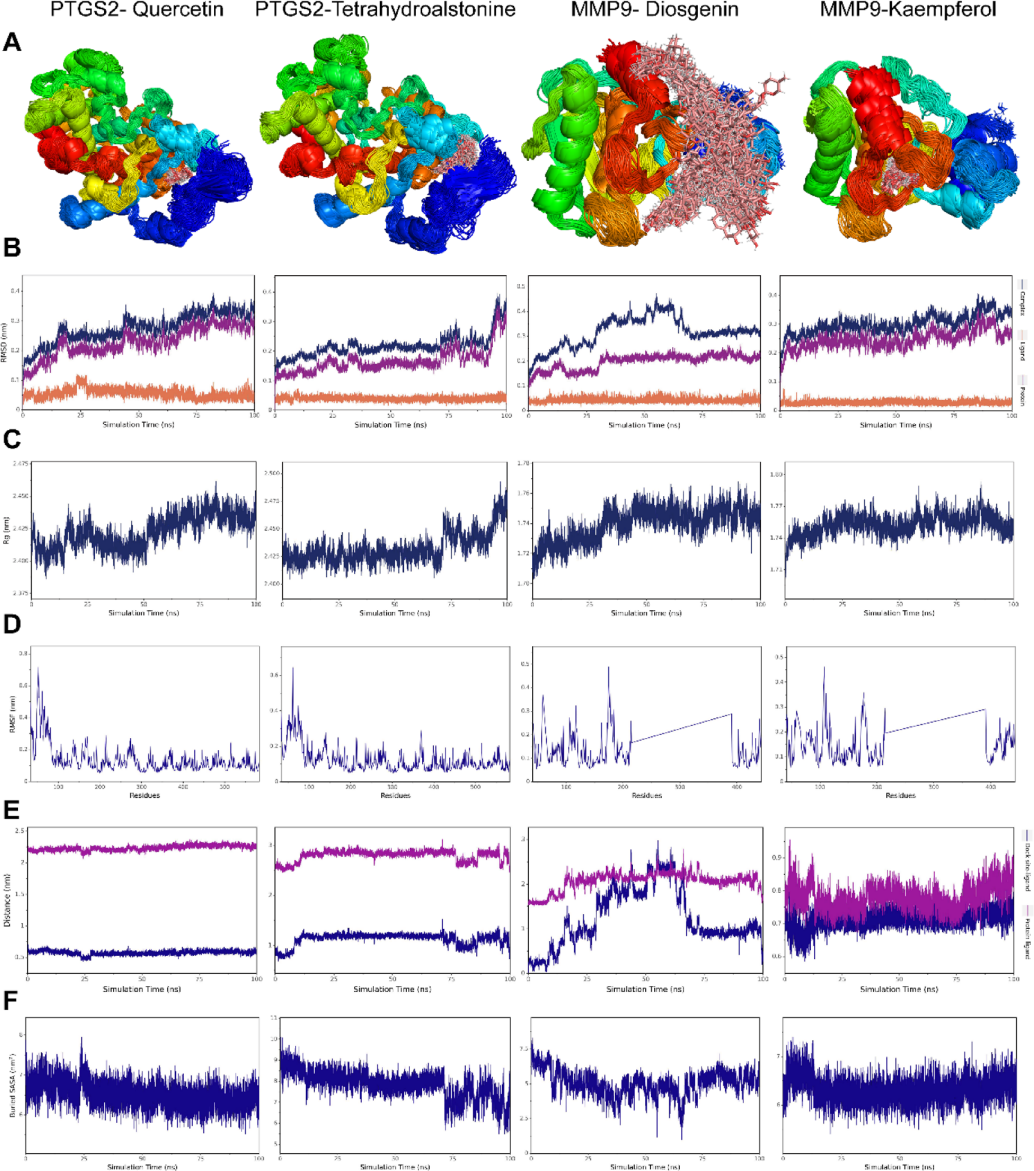
Stability analysis of complex. (**A**) Simulated conformational superposition. (**B**) RMSD Analysis of Complex, Protein and Ligand. (**C**) Rg of complex. (**D**) RMSF of protein in complex. (**E**) Distance between small molecules and protein binding sites. (F) Buried SASA between small molecules and proteins.

We analyzed the simulated trajectories by superimposing the simulated conformations (Fig. 6A). The degree of small molecule stacking was higher in PTGS2-Quercetin, PTGS2-Tetrahydroalstonine, and MMP9-Kaempferol, while the small molecule stacking conformation of MMP9-Diosgenin was more dispersed, however, this small molecule was consistently bound in and around the initial binding site. To assess the stability of the small molecule-protein binding, we examined various parameters including RMSD, Radius of Gyration (Rg), Root mean square fluctuation (RMSF), centroid evolution, and the buried solvent-accessible surface area (Buried SASA) of the small molecules buried in the protein. Among these parameters, Rg can describe overall structural changes and characterize the compactness of the protein structure. Larger changes in Rg indicate a more expanded system. RMSF can indicate the flexibility of amino acid residues in the protein. Centroid evolution and Buried SASA can indicate the binding state of small molecules to the protein.

The results demonstrated that the structures of the four complexes eventually reached stability (Fig. 6B–F). It is worth noting that the RMSD of the MMP9-Diosgenin complex gradually stabilized after 75 ns, while the RMSD of the protein remained largely stable after 30 ns. This suggests that the fluctuation in the RMSD of the complex was primarily caused by the small molecule. However, the conformation of the small molecule remained near the initial binding site throughout the 100 ns simulation, indicating that the small molecule remained bound to the protein. In the case of PTGS2-Tetrahydroalstonine, the Rg gradually increased after 70 ns, and the Buried SASA showed a slight decrease. This was caused by the gradual detachment of the protein edge structure from the protein main structure, resulting in an increase in the overall radius of gyration of the complex and a decrease in the contact area between the small molecule and the protein. However, this did not affect the binding of the small molecule to the protein. The distances between the small molecules and the protein centers, as well as the distances between the small molecules and the initial binding sites, remained stable for all four complexes, with only minor fluctuations. Overall, these findings indicate that the small molecule-protein complexes achieved stability during the simulation period, and the small molecules remained bound to their respective protein targets.

#### Analysis of hydrogen bonding interactions of small molecules with proteins

**Fig. 7:**
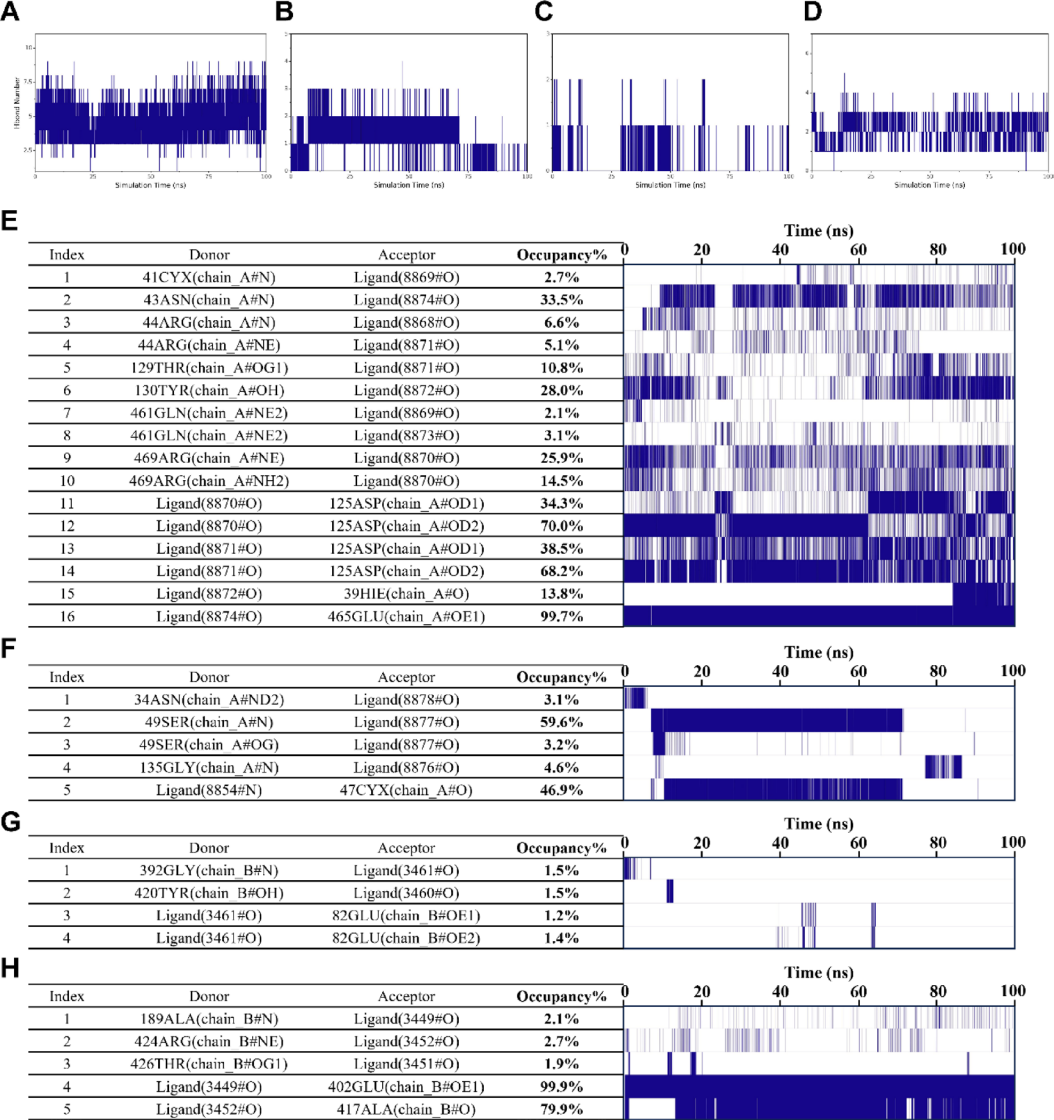
Hydrogen bond number of (**A**) PTGS2-Quercetin, (**B**) PTGS2-Tetrahydroalstonine, (**C**) MMP9- Diosgenin, (**D**) MMP9-Kaempferol. Hydrogen bond frequency of (**E**) PTGS2-Quercetin, (**F**) PTGS2-Tetrahydroalstonine, (**G**) MMP9-Diosgenin, (**H**) MMP9-Kaempferol.

Hydrogen bonding plays a crucial role in protein–ligand binding as it is a significant force of interaction. These bonds are associated with electrostatic interactions and can indicate the strength of these interactions. Our results revealed that the number of hydrogen bonds between the small molecules of PTGS2-Quercetin and the proteins fluctuated mainly between 3 and 6. In contrast, the number of hydrogen bonds between the small molecules of PTGS2-Tetrahydroalstonine and the proteins was lower, fluctuating primarily between 1 and 2. Similarly, the number of hydrogen bonds between the small molecules of MMP9-Diosgenin and the proteins was also lower. For MMP9-Kaempferol, the number of hydrogen bonds between the small molecules and proteins mainly fluctuated between 1 and 3 (Fig. 7A–D).

To gain further insights into the residues in the protein that form hydrogen bonds with small molecules and to assess the stability of these hydrogen bonds, we analyzed the occupancy of the hydrogen bonds formed between the small molecules and the protein. As shown in (Fig. 7E–H), with the acceptor, donor, and occupancy of the hydrogen bonding pairs displayed on the left side. On the right side, the frequency of hydrogen bond formation is depicted, with the denseness of the lines representing the frequency of hydrogen bond formation. In the case of PTGS2-Quercetin and MMP9-Kaempferol, relatively stable hydrogen bonding pairs were observed between the small molecules and the proteins. However, for PTGS2-Tetrahydroalstonine, stable hydrogen bonding pairs were present only during the initial 0–70 ns period. In contrast, MMP9-Diosgenin did not exhibit any stable hydrogen bonding between the small molecule and the protein.

#### Analysis of small molecule-protein binding interactions

**Fig. 8:**
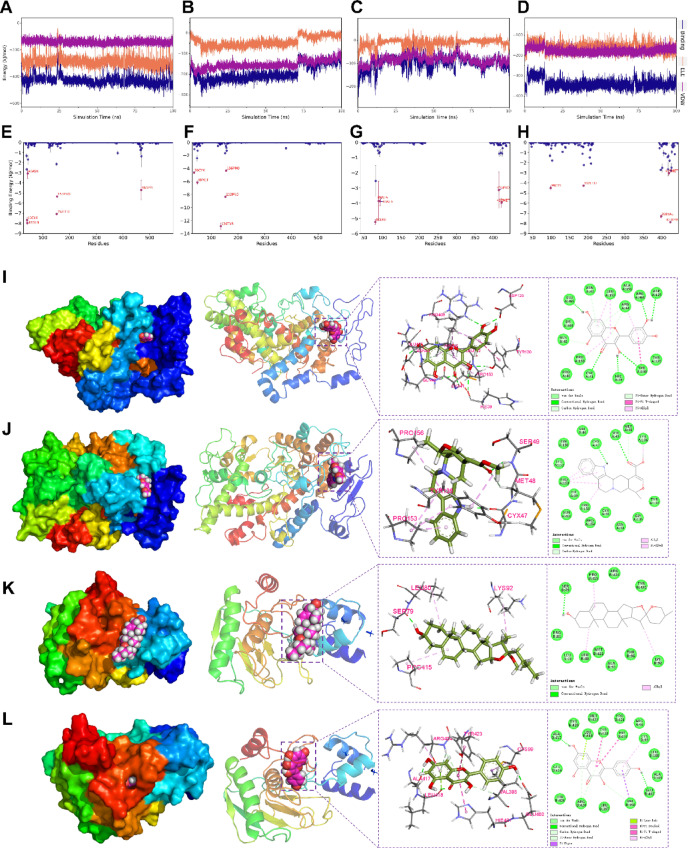
Binding energy between small molecules and proteins: (**A**) PTGS2-Quercetin, (**B**) PTGS2-Tetrahydroalstonine, (**C**) MMP9- Diosgenin, (**D**) MMP9-Kaempferol. Amino acid binding energy contribution of (**E**) PTGS2-Quercetin, (**F**) PTGS2-Tetrahydroalstonine, (**G**) MMP9-Diosgenin, (**H**) MMP9-Kaempferol. Interaction between proteins and small molecules: (**I**) PTGS2-Quercetin, (**J**) PTGS2-Tetrahydroalstonine, (**K**) MMP9-Diosgenin, (**L**) MMP9-Kaempferol.

The van der Waals and electrostatic interaction forces between the small molecules of the complex and the protein were calculated without considering solvation, and the change in the binding force during the simulation was analyzed. Here, VDW represents the van der Waals force and hydrophobic interaction, ELE represents the electrostatic interaction, and Binding is the sum of VDW and ELE, serving as a representation of the binding energy between small molecules and proteins without considering the solvation effect. The results showed that VDW and ELE in PTGS2-Quercetin and MMP9-Kaempferol both remained stable, indicating that the binding between small molecules and proteins was consistent. Similarly, VDW and ELE in PTGS2-Tetrahydroalstonine remained relatively stable between 0 and 70 ns, suggesting stability in the binding between small molecules and proteins during this period. In the case of MMP9-Diosgenin, VDW and ELE gradually stabilized after 70 ns, indicating a gradual stabilization of the binding between the small molecules and proteins (Fig. 8A–D).

Taking into account the solvation energy, the RMSD, Rg, Distance, BuriedSASA, and interaction energy are combined, and the trajectories of the complexes in the steady state are selected and calculated using the Molecular Mechanics-Poisson Boltzmann Surface Area (MM-PBSA) method to obtain the binding energy-related terms. The results show that both ΔEvdw and ΔEele in PTGS2-Quercetin are higher than ΔEnonpol. This suggests that van der Waals force interactions and electrostatic interactions play a major role, while hydrophobic interactions play a minor role in the composition of the binding energies. In the PTGS2-Tetrahydroalstonine, ΔEvdw is higher than ΔEele, the former is 7.5 times higher than the latter, and ΔEele is similar to ΔEnonpol. Therefore, van der Waals interaction plays a major role, while electrostatic and hydrophobic interactions play a minor role in the composition of the binding energy. For MMP9-Diosgenin, ΔEvdw is higher than ΔEele, with the former being 6.7 times that of ΔEnonpol, and both being higher than ΔEele. Hence, van der Waals interactions play a major role, while electrostatic interactions play a minor role, and hydrophobic interactions play a complementary role in the composition of binding energies. In the MMP9-Kaempferol, ΔEvdw is higher than ΔEele, with the former being 2.2 times that of the latter, and both being higher than ΔEnele. Thus, van der Waals interactions play a major role, electrostatic interactions play a minor role, and hydrophobic interactions play a complementary role in the composition of binding energies (Table 5).

**Table 5:** Binding energy and composition of MMPBSA in steady state. (kJ/mol).

Parameters	PTGS2		MMP9	
	Quercetin	Tetrahydroalstonine	Diosgenin	Kaempferol
ΔE_vdw_	-156.695 ± 3.643	-173.13 ± 1.755	-101.967 ± 1.899	-180.895 ± 2.209
ΔE_vdw_	-151.295 ± 1.959	-23.365 ± 1.279	-2.399 ± 1.938	-81.171 ± 1.517
ΔE_pol_	276.672 ± 1.057	99.595 ± 0.641	36.118 ± 4.526	207.512 ± 1.208
ΔE_nonpol_	-16.894 ± 0.086	-21.328 ± 0.045	-15.057 ± 0.206	-16.683 ± 0.019
ΔE_MMPBSA_	-48.214 ± 2.017	-118.228 ± 1.387	-83.305 ± 2.308	-71.236 ± 1.871
-TΔS	29.72 ± 6.579	16.399 ± 1.704	15.735 ± 2.356	13.316 ± 1.563
ΔG_bind_*	-18.494 ± 5.538	-101.829 ± 3.084	-67.569 ± 4.652	-57.92 ± 0.453

The results of ΔEMMPBSA indicated a high binding energy and affinity between the small molecules of the four complexes and the proteins. To further understand the contribution of each amino acid to the overall binding energy and evaluate the important amino acids in the proteins, the ΔEMMPBSA was decomposed. From the figure, it is evident that the key amino acids for binding the small molecules in the PTGS2-quercetin protein include GLN-42 and CYX-41, etc.; the key amino acids for binding the small molecules in the PTGS2-Tetrahydroalstonine has TYR-136 and PRO-153, etc.; MMP9-Diosgenin has LEU-80 and MET-422, etc.; MMP9-Kaempferol has TYR-432 and VAL-398, etc. (Fig. 8E–H).

Further resolving their structures and interactions, PTGS2-Quercetin was found to have selected the conformation at the end of the simulation, where amino acids GLN-465, ARG-469, ASP-125, TYR-130, HIE-39, and CYX-41 in the protein formed hydrogen bonds with the small molecule. Additionally, TYR-130, LEU-152, ARG-44, and PRO-153 formed Pi-Pi T-shaped and Pi-Alkyl hydrophobic interactions with the small molecules, while amino acids ALA-151 and PRO-40 formed van der Waals force interactions with the small molecules. In the PTGS2-Tetrahydroalstonine, the conformation at 55 ns was selected, and amino acids SER-49 and CYX-47 in the protein formed hydrogen bonds with the small molecules. Furthermore, PRO-153 and PRO-156 formed Alkyl and Pi-Alkyl hydrophobic interactions with the small molecules, while amino acids such as VAL-46 and ASN-34 formed van der Waals interactions with the small molecules. For MMP9-Diosgenin, the conformation at the end of the simulation was selected, and amino acids SER-779 in the protein formed hydrogen bonds with the small molecules. Additionally, amino acids PRO-415, LEU-80, and LYS-92 formed Alkyl hydrophobic interactions with the small molecules, and amino acids ARG-424 and ALA-93 formed van der Waals force interactions with the small molecules. Lastly, MMP9-Kaempferol was found to have selected the end-of-simulation conformation, in which amino acids GLU-402 in the protein formed hydrogen bonds with the small molecules. Furthermore, VAL-398, LEU-418, TYR-423, HIE-401, and CYS-99 formed Pi-Sigma, Pi-Lone Pair, Pi-Pi Stacked, Pi-Pi T-shaped, and Pi-Alkyl hydrophobic interactions with the small molecules, while MET-422, LEU-397, and other amino acids formed van der Waals force interactions with the small molecules (Fig. 8I–L).

In summary, the small molecules of PTGS2-Quercetin, PTGS2-Tetrahydroalstonine, MMP9-Diosgenin and MMP9-Kaempferol were all stably bound to the proteins with high binding energy and affinity.

## Discussion

In this retrospective study of 80 pediatric ASD cases, LW demonstrated significant improvement in core ASD symptoms, as evidenced by marked reductions in ABC and CARS scale scores. Notably, we observed an age-dependent treatment effect, with younger children exhibiting greater therapeutic responsiveness and more pronounced symptom improvement. Furthermore, ASD typically manifests in early childhood, with related symptoms observable before the age of 3. However, many children with ASD are not identified and treated until school age, possibly because the symptoms of ASD are not obvious in early childhood or are misunderstood as other developmental issues, leading to delayed diagnosis and treatment^19^. Children with ASD often exhibit characteristics of delayed speech and delayed walking during their growth and development. Delayed speech and poor comprehension may be early age-specific markers of ASD^20^. Delayed walking and slower walking speed are related to lower scores in the development of communication, motor skills, and adaptive functions^21^. As ASD children enter school age and adolescence, symptoms in some children may worsen with age, possibly due to the need to face more complex social interactions and learning environments. E/I ratio can be estimated from resting-state functional magnetic resonance imaging (fMRI) using the Hurst exponent, H. Studies have shown that the ventromedial prefrontal cortex (vmPFC) H in the cerebral cortex plays an important role in emotion regulation, social cognition, and decision-making, and the vmPFC H in children with ASD decreases with age, reflecting an increase in the E/I ratio, which may be related to the worsening of ASD symptoms^22^. Therefore, early intervention and continuous support therapy for children with ASD are crucial to help them better cope with the challenges of growth and development.

LW exhibits multiple neuropharmacological activities. It has been found to effectively treat Alzheimer’s disease by modulating immune cell infiltration in the brain^23^, increasing autophagy in hippocampal neurons, reducing Aβ precipitation, and inhibiting the activation of pro-inflammatory microglia and astrocytes^14^. Additionally, it can regulate lipid metabolism and oxidative stress by restoring the homeostasis of the microbiota-gut-brain axis, thereby enhancing cognitive function in aged mice^15^. Our study found that LW has a good therapeutic effect on children with ASD, and the younger the age, the better the effect. Among the 10 main active ingredients of LW that have been identified, Quercetin has been found to possess antioxidant properties that are beneficial in the treatment of neurological disorders, making it a potential drug for the treatment of ASD^24^. Stigmasterol and Kaempferol have been shown to promote neurogenesis and synaptogenesis^25,26^. Additionally, other active ingredients in LW have demonstrated various neuroprotective pharmacological effects.

The GO enrichment analysis of potential targets of LW indicates their involvement in a variety of biological processes, cellular composition, and molecular functions. Specifically, hormone^27^, synaptic membrane^28^, and postsynaptic membrane^29^ have been identified as closely related to ASD. KEGG pathway analysis revealed that among the signaling pathways mainly involved in disease target genes, PI3K-Akt signaling pathway^30^, MAPK signaling pathway^31^, AGE-RAGE signaling pathway^32^, and Toxoplasmosis^33^ are considered to be associated with ASD. Among the key target genes of LW for ASD, AKT1 is associated with social activities^34^, TNF, PTGS2, MMP9, and IL1β are linked to chronic inflammation in the brain^35–37^, and GSK3β is associated with synaptic remodeling^38^. The abnormal function of these genes may play an important role in the pathogenesis of ASD.

Similarly, our analysis of microarray data revealed that the expression of PTGS2 and MMP9 genes is upregulated in ASD, which aligns with the therapeutic targets mentioned earlier. The PTGS2 gene encodes an enzyme called cyclooxygenase 2 (COX2), which plays a crucial role in neurodevelopmental mechanisms and other important processes. In the healthy brain, COX2 converts arachidonic acid (AA) released from cell membranes into prostaglandin E2 (PGE2) and other prostaglandin metabolites^39^. PGE2 is a major lipid molecule in the nervous system and has been linked to abnormal signaling in ASD^40,41^. Studies have shown that COX2-deficient mice exhibit autistic-like behaviors, including hyperactivity, anxiety, repetitive behaviors, motor deficits, and social abnormalities, along with impaired dendritic morphology and dendritic spines. These abnormalities are more pronounced in male COX2-deficient mice, which is consistent with existing studies on gender differences in children with ASD^39,42^. Additionally, PTGS2 is closely associated with neuroinflammation and regulates inflammatory responses in neuronal synapses. Some children with ASD exhibit an innate neuroinflammatory response accompanied by increased expression and activity of COX2 encoded by the PTGS2 gene^43^, which can serve as a diagnostic marker for ASD^44^. MMP9 is widely present in the brain and is involved in neuronal development and synaptic plasticity. It can be produced by various cells such as neurons, microglia, and astrocytes, and plays a role in brain learning and memory processes^45^. However, excessive activity of MMP-9 can disrupt proper learning, memory, and synaptic plasticity^46,47^. In a study where maternal immune activation induced ASD in offspring, elevated MMP9 expression was observed in neurons of the prefrontal cortex of ASD mice, which positively correlated with the level of inflammation. Herbal treatment was able to reverse this change^36^, and similar results were found in the hippocampus of children with ASD^48^. Therefore, MMP9 holds potential as a biomarker or therapeutic target.

Molecular dynamics simulations are commonly employed to model the movements and interactions of atoms and molecules over time, providing insights into the structures, dynamic behaviors, and interactions of biomolecules. Through these simulations and subsequent binding free energy analyses, the functions and properties of biomolecules can be studied and understood. In the case of PTGS2-Quercetin, PTGS2-Tetrahydroalstonine, MMP9-Diosgenin, and MMP9-Kaempferol, the small and medium molecule active compounds were found to be stably bound to the proteins with high binding energy and affinity. Previous studies have confirmed that Quercetin can target the PTGS2 gene to inhibit neuronal iron death in mice^49^, and Diosgenin and Kaempferol can reduce MMP9 levels and inhibit neuroinflammatory responses in neurological diseases^50,51^. Therefore, these small molecule compounds have the potential to bind to the active site of proteins and exert an inhibitory effect. They can be considered strong and stable inhibitors binding within the protein-binding pocket, warranting further investigation for the design and development of inhibitors targeting specific proteins. Moreover, with further chemical modification and optimization, these compounds may evolve into potential drugs for the treatment of ASD, offering new therapeutic options.

## Conclusion

LW has been shown to have a good therapeutic effect on children with ASD. Its active ingredients can act on multiple targets through various signaling pathways, thereby treating ASD. The complexes formed by PTGS2-Quercetin, PTGS2-Tetrahydroalstonine, MMP9-Diosgenin, and MMP9-Kaempferol were found to be stable and exhibited a strong affinity for ASD. This study provides a theoretical basis for the rational clinical application of LW and offers a new approach for treating ASD. However, there are some limitations in this study. The publicly available databases and datasets used may have certain limitations, and it is not possible to consider all biological factors comprehensively. Furthermore, experimental validation is necessary to confirm the accuracy and reliability of the results obtained through these methods.

## Supplementary Information

Below is the link to the electronic supplementary material.

Supplementary Material 1

Supplementary Material 2

## Data Availability

The authors confirm that the data supporting the findings of this study are available within the article [and/or] its supplementary materials.

## Abbreviations

LW: Liuwei Dihuang decoction
ASD: Autism spectrum disorders
MD: Molecular dynamics
ABC: Autism Behavior Checklist
CARS: Childhood Autism Rating Scale
SDH: Rehmannia rhizome
SZY: Cornus
SY: Common yam rhizome
FL: Poria
DP: Tree peony bark
ZX: Alisma rhizome
TCMSP: Traditional Chinese Medicine Systematic Pharmacology Database
PPI: Protein–Protein Interaction
BC: Between Centrality
CC: Closeness Centrality
GO: Gene Ontology
KEGG: Kyoto Encyclopedia of Genes and Genomes
RMSD: Root mean square deviation
Rg: Radius of Gyration
RMSF: Root mean square fluctuation
PTGS2: Prostaglandin G/H synthase 2
MMP9: Matrix metalloproteinase-9
COX2: Cyclooxygenase 2
PGE2: Prostaglandin E2
