# Supplementary material for: Unveiling the multitarget mechanism of Liuwei Dihuang decoction in autism spectrum disorder via network pharmacology and molecular docking
Source: Sci Rep. 2025 Nov 25;15:44559. doi: 10.1038/s41598-025-28204-1 (PMC12738649; doi:10.1038/s41598-025-28204-1)
Supplement: Supplementary file 1 — Supplementary Material 1 [file 41598_2025_28204_MOESM1_ESM.docx]

Supplementary table. Active compounds of LW

| MOL ID | Molecule Name | OB (%) | DL | origin |
| --- | --- | --- | --- | --- |
| MOL000449 | Stigmasterol | 43.83 | 0.76 | SDH、SZY、SY |
| MOL000359 | sitosterol | 36.91 | 0.75 | SDH、SZY、DP、ZX |
| MOL005531 | Telocinobufagin | 69.99 | 0.79 | SZY |
| MOL001495 | Ethyl linolenate | 46.1 | 0.2 | SZY |
| MOL002879 | Diop | 43.59 | 0.39 | SZY |
| MOL001494 | Mandenol | 42 | 0.19 | SZY |
| MOL005503 | Cornudentanone | 39.66 | 0.33 | SZY |
| MOL001771 | poriferast-5-en-3beta-ol | 36.91 | 0.75 | SZY |
| MOL000358 | beta-sitosterol | 36.91 | 0.75 | SZY |
| MOL005530 | Hydroxygenkwanin | 36.47 | 0.27 | SZY |
| MOL005481 | 2,6,10,14,18-pentamethylicosa-2,6,10,14,18-pentaene | 33.4 | 0.24 | SZY |
| MOL008457 | Tetrahydroalstonine | 32.42 | 0.81 | SZY |
| MOL002883 | Ethyl oleate (NF) | 32.4 | 0.19 | SZY |
| MOL003137 | Leucanthoside | 32.12 | 0.78 | SZY |
| MOL000546 | diosgenin | 80.88 | 0.81 | SY |
| MOL001736 | (-)-taxifolin | 60.51 | 0.27 | SY |
| MOL005430 | hancinone C | 59.05 | 0.39 | SY |
| MOL000322 | Kadsurenone | 54.72 | 0.38 | SY |
| MOL005465 | AIDS180907 | 45.33 | 0.77 | SY |
| MOL005440 | Isofucosterol | 43.78 | 0.76 | SY |
| MOL000953 | CLR | 37.87 | 0.68 | SY |
| MOL005435 | 24-Methylcholest-5-enyl-3belta-O-glucopyranoside_qt | 37.58 | 0.72 | SY |
| MOL005438 | campesterol | 37.58 | 0.71 | SY |
| MOL005458 | Dioscoreside C_qt | 36.38 | 0.87 | SY |
| MOL001559 | piperlonguminine | 30.71 | 0.18 | SY |
| MOL000273 | (2R)-2-[(3S,5R,10S,13R,14R,16R,17R)-3,16-dihydroxy-4,4,10,13,14-pentamethyl-2,3,5,6,12,15,16,17-octahydro-1H-cyclopenta[a]phenanthren-17-yl]-6-methylhept-5-enoic acid | 30.93 | 0.81 | FL |
| MOL000275 | trametenolic acid | 38.71 | 0.8 | FL |
| MOL000279 | Cerevisterol | 37.96 | 0.77 | FL |
| MOL000282 | ergosta-7,22E-dien-3beta-ol | 43.51 | 0.72 | FL |
| MOL000283 | Ergosterol peroxide | 40.36 | 0.81 | FL |
| MOL000296 | hederagenin | 36.91 | 0.75 | FL |
| MOL000211 | Mairin | 55.38 | 0.78 | DP |
| MOL000422 | kaempferol | 41.88 | 0.24 | DP |
| MOL000492 | (+)-catechin | 54.83 | 0.24 | DP |
| MOL007374 | 5-[[5-(4-methoxyphenyl)-2-furyl]methylene]barbituric acid | 43.44 | 0.3 | DP |
| MOL000098 | quercetin | 46.43 | 0.28 | DP |
| MOL002464 | 1-Monolinolein | 37.18 | 0.3 | ZX |
| MOL000862 | [(1S,3R)-1-[(2R)-3,3-dimethyloxiran-2-yl]-3-[(5R,8S,9S,10S,11S,14R)-11-hydroxy-4,4,8,10,14-pentamethyl-3-oxo-1,2,5,6,7,9,11,12,15,16-decahydrocyclopenta[a]phenanthren-17-yl]butyl] acetate | 35.58 | 0.81 | ZX |
| MOL000856 | alisol C monoacetate | 33.06 | 0.83 | ZX |
| MOL000853 | alisol B | 36.76 | 0.82 | ZX |
| MOL000849 | 16β-methoxyalisol B monoacetate | 32.43 | 0.77 | ZX |
